# Editorial: Membrane Processes in Erythroid Development and Red Cell Life Time

**DOI:** 10.3389/fphys.2021.655117

**Published:** 2021-02-18

**Authors:** Giampaolo Minetti, Anna Rita Migliaccio, Eitan Fibach

**Affiliations:** ^1^Laboratories of Biochemistry, Department of Biology and Biotechnology “L. Spallanzani,” University of Pavia, Pavia, Italy; ^2^Biomedical and Neuromotorial Sciences, Alma Mater Studiorum University, Bologna, Italy; ^3^Myeloproliferative Neoplasm Research Consortium, New York, NY, United States; ^4^Department of Hematology, Hadassah Medical Center, Jerusalem, Israel

**Keywords:** cultured red blood cells, reticulocyte maturation, red blood cell aging, membrane rafts, exosomes, vesiculation, autophagy, cholesterol

Despite the intense research on erythroid development of the past years, many questions are still open concerning the red blood cell (RBC) life cycle: the maturation of the reticulocyte (retic), a process that for its complexity is now defined terminal erythroid maturation, and RBC senescence and death. Although for subject and methodology the papers included in this Research Topic are very diverse, they all represent a snap-shot of the most relevant questions of RBC physiology being addressed at the moment.

When published, in 1986, “The Reticulocyte,” a book by Samuel Mitja Rapoport, represented a thorough perusal of the currently available literature and own work, set the basis for, and stimulated the systematic investigations that followed. The book was seminal, one can find there the seeds of all the important avenues that retic investigators undertook in the years to come. The idea that ATP-ubiquitin-dependent processes control the breakdown of selected retic proteins was there (Ciechanover's discovery of ubiquitin in rabbit retics dated back to only a few years earlier). So were the concepts, although still in their embryonic stage, of autophagic mechanisms for the degradation of mitochondria, and of vesicular trafficking for the selective release of plasma membrane proteins (e.g., the transferrin receptor, TfR) and lipids in the form of vesicles (now called exosomes). In the years to follow, significant advancements have been made in the understanding of erythroid and retic maturation in the marrow. Among other issues that have remained rather unexplored is the question of the relation between the maturation of retics and the aging of RBCs. The importance of this question was stressed by Rapoport in the concluding remarks of his book (Rapoport, 1986). Understanding of the mechanisms underlying retic production, release, and maturation is a key to successful production of RBCs from discarded (sometimes molecularly engineered) stem cells for transfusion purposes. The perfection of the techniques for *ex vivo* production of retics may also be useful for research, for instance to obtain retics from other mammals, such as the horse, which have no circulating retics (Kämpf et al.). This brings us to articles in this series that reveal new aspects of retic maturation in health and disease.

Retics are generated by enucleation of the orthochromatic erythroblast and go through a maturation process in which four stages have been identified: from R1 retics in the bone marrow, with detectable RNA and TfR, through the circulating R2 and R3, to the most mature R4, having no TfR left in the membrane and only residual RNA inside. One of the main unsolved problems is the terminal maturation of circulating, R2 retics. Much more is known regarding the enucleation and R1 maturation since these processes are largely spontaneous and can be recapitulated *in vitro*, albeit less efficiently than *in vivo*. These highly complex events are prone to perturbation by defects in any of the intracellular processes with which they are coordinated. This is nicely illustrated by the article of Flatt et al., who used a loss-of-function approach to show how, in subjects with Southeast Asian Ovalocytosis band 3 variant, retic maturation is deranged from the very stage of enucleation on to the retic maturation, due to band 3 misfolding that impacts on the internal quality control, vesicular trafficking and autophagy mechanisms.

Terminal maturation of retics is mostly played at the plasma membrane. Evolutionary pressure has evidently selected for the progressive biogenesis and stabilization of a unique lipid bilayer-membrane skeleton complex that characterizes the mammalian biconcave discocyte. In addition to its physiological advantage, membrane stability and robustness render mature RBCs suitable for biotechnological applications and *ex vivo* manipulations that make them drug-carriers, or cellular therapeutics, as illustrated in the article by Rossi et al.

To reduce its size, the R1 retic releases significant amounts of its plasma membrane and most of the TfR as exosomes, which also appear to be enriched in membrane rafts; autophagy operates the dismantling of mitochondria and other organelles, and ATP/ubiquitin/proteasome-mediated proteolysis takes care of the removal of selected unneeded intracellular proteins. Different investigators contributing to this Research Topic conclude that membrane lipids play a key role in the proliferation and maturation process of erythroid precursors (Zingariello et al., Bernecher et al., Minetti et al.). Cultured, but also *in vivo* produced, retics suffer from membrane stability problems, possibly because of the lower deformability of a more spherical cell and a still immature connectivity between the bilayer and the membrane skeleton. Yet, from Bernecker et al.'s article, we learn that cultured retics display a severe cholesterol deficiency, which the authors directly connect with an even higher osmotic fragility displayed by these cells with respect to natural retics. In fact, the defect can be almost completely corrected by lipid supplementation during culturing, which brings the cholesterol content in the membrane to near normal levels. Conversely, cholesterol depletion, with methyl-ß-cyclodextrin, renders normal mature RBCs osmotically fragile.

Zingariello et al. also discuss in their paper the cholesterol deficiency of cultured retics and highlight the importance of lipid composition of the culture medium that, by optimizing cholesterol bioavailability, increases both the yield and the maturation of retics. This sheds light on the long-known problem that dexamethasone (a glucocorticoid receptor agonist added to culture media for sustaining the first proliferative phase of erythroid development) decreases the efficiency of enucleation *in vitro*. It was previously observed that the enucleation rate dramatically increased when cultured human erythroblasts were injected into an immune-deficient host (mouse). This suggested that dexametasone induced a defect in the composition of the membrane, in particular of the lipid moiety, which could be reverted once the cells were infused into the host, from which they evidently acquired the missing component(s). The present study (Zingariello et al.) indeed shows that supplementation of the culture medium with the proper formulation of light-density (LDL) and very-light-density (VLDL) lipoprotein, two physiological cholesterol carriers, increases the proliferation rate and prevents the autophagic death of erythroid precursors.

Yet another article from Minetti et al., reports on the profound rearrangement of membrane lipids in the maturation of circulating R2 retics. Starting from the established fact that membrane rafts are selectively lost from the membrane of maturing R1 retics (being enriched in the membrane of the released exosomes), the authors hypothesize that rafts may also be lost in the maturation of R2 retics. However, by comparing the lipid composition of pure R2 retics with that of the membrane of mature RBCs from the same donors, the authors find the exact opposite. Those lipids that are typical of membrane rafts, cholesterol and sphingolipids, are actually enriched in the membrane of mature RBCs. Protein markers of rafts behave differently depending on protein type: whereas flotillins appear to be lost by R2 retics, stomatin maintains the same levels. The conclusions that authors draw from these data are twofold. First, membrane rafts must be heterogeneous, with different raft classes bearing different protein composition. The heterogeneous nature of rafts cannot be appreciated when they are all together isolated as detergent-resistant-membrane. Second, the finding fits perfectly with the concept, first proposed by this research group, that rafts serve the purpose of additional anchoring sites for the bilayer to the membrane skeleton in mature RBCs. The association is probably mediated by a direct lipid-skeleton interaction, as no protein anchor and the corresponding anchoring site(s) in the membrane skeleton have been identified so far (band 3 and glycophorins are completely absent in membrane rafts).

Another result of this work is that band 3 and spectrin are partially lost as R2 retics mature. This is not trivial, since the exosomes with which presumably most of the excess membrane is lost by R1 retics are completely devoid of band 3, glycophorins and membrane skeletal proteins. This also shows that the two phases of retic maturation occur by two completely different mechanisms, where R1 maturation is largely spontaneous whereas R2 maturation is not; it requires the intervention of other organs (spleen, liver, endothelium). Moreover, the hypothesized continuum (Minetti et al., 2018) from retic maturation to RBC senescence that proceeds from large irregularly shaped retics to progressively smaller and yet still biconcave discocytes to the last day of RBC life, still holds, although it appears to be sustained by different mechanisms.

Concerning the latter issue, a connection can now be made between retic maturation and RBC aging. The same research group has recently observed that, contrary to what is generally believed, i.e., that human RBCs lose membrane, as they age, in the form of spectrin-free vesicles, they may actually lose portions of lipid bilayer and membrane skeleton simultaneously (Ciana et al., 2017). If confirmed, this result would again point to non-spontaneous processes driving the changes that RBCs undergo from their R2 retic stage to the last day of RBC circulatory life. The discussion around the issue of vesiculation of RBCs *in vivo* has been boosted by the theoretical elaboration by Asaro et al. They propose that under the particular flow conditions that RBCs face when they transit from the splenic red pulp through the slits in the endothelium that lines the venous sinuses, vesicles may be released by a process of “infolding.” Interestingly, the model can be tuned to predict the formation of both skeleton-free and skeleton-containing vesicles. A more refined model from the same group includes an active role possibly played by splenic endothelial cells in facilitating/inducing the release of vesicles via tethering under the peculiar splenic flow conditions (Asaro et al., 2020). Given the plethora of interactions that RBCs experience with other cell types, and that are extensively reviewed in the article by Pretini et al., this aspect cannot be excluded from theoretical models. Attempts to reproduce a mechanical splenic action to induce maturation of cultured retics to biconcave discocytes (Moura et al., 2018), have so far failed. Yet, it would be of paramount importance to experimentally test the theoretical model described above to verify whether a properly set microfluidic system could be tuned to actually produce vesicles with or without membrane-skeletal proteins. If sufficient amounts of vesicles could be produced with this method, they could be easily probed for the presence or absence of membrane skeleton proteins (e.g., spectrin). Along this line, it must be also admitted that a purely mechanical remodeling may not work on cultured retics because they are different from their native counterpart. That these retics have impaired osmotic resistance, linked to cholesterol deficiency in their membrane, has been shown only recently by the above-mentioned work of Bernecker et al. Finding better culturing conditions would be the starting point for verifying if and to what extent shear forces could be sufficient to convert cultured retics into discocytes.

As said, solving the problem of how circulating retics or mature RBCs lose membrane is important for shedding light on the properties of the senescent RBC and the mechanisms of its clearance from the circulation (Kaestner and Minetti, 2017). A strain of mice is described, which express a red fluorescent protein in the erythroid lineage, producing red fluorescent RBCs. This technique proved useful in applications such as intravital microscopy, 3D-shape analysis, and transfusion experiments, with the possibility, therefore, to study RBC aging *in vivo*. Concerning this subject, Bernhardt et al. draw attention to the two vexed issues: is there any increase in intracellular free Ca2^+^ and in phosphatidylserine (PS) exposure in old RBCs? This topic is paradigmatic of situations where different results are obtained when working with RBC in bulk or with single cells, or populations of cells selected according to e.g., cell age. Moreover, intrinsic difficulties in the methods of measuring intracellular free Ca^2+^ have rendered even more difficult to reach consensus. Authors try to reconcile conflicting data from two sets of experiments aimed at correlating RBC density (age) with PS exposure. They conclude, on the one hand, that conflicting results make the increased intracellular Ca^2+^ with aging elusive. On the other hand, more consistent reports exist that allow concluding that PS exposure does not change with cell age. Thus, probably, the variation of the free intracellular Ca^2+^ concentration does not exceed 10 μM, which appears to be the threshold for scramblase activation (inducing PS exposure) and flippase inactivation (blocking the energy-dependent translocation of PS from the outer to inner membrane leaflet) (Bernhardt et al.).

As discussed throughout the Research Topic, membrane shedding, in the form of microvesicles, plays a key role in erythroid cells. Partial disturbance of the membrane-cytoskeleton linkage and increased intracellular Ca^2+^ content are considered to be mechanisms underlying the process, but it is questionable whether they constitute the primary initiating steps. In a review by Fibach, he summaries some data-based and hypothetical possibilities that await experimental confirmation regarding the interaction between membrane shedding and the redox system. The latter depends on the equilibrium between oxidants and antioxidants—excess oxidants results in oxidative stress, which affects many cellular components, including the membrane. Oxidative stress may initiate membrane shedding by indirect effect on the membrane-cytoskeleton and the Ca^2+^ content. RBCs undergo changes in both their redox system and membrane shedding throughout their life—from birth—their production in the bone marrow, to death—aging in the peripheral blood and clearance in the reticuloendothelial system, serving specific roles at each stage. Both processes are disturbed in diseases affecting RBCs, such as the hereditary and acquired hemolytic anemias (i.e., thalassemia, sickle cell anemia, and autoimmune hemolytic anemia). Antioxidants may represent an important therapeutic modality.

Together with articles collected in other topics on RBCs (Kaestner and Bogdanova, 2018), we hope that this Research Topic, focused on the membrane of retics and RBCs, will contribute to the wealth of knowledge that has accumulated over the past years in the Red Blood Cell Physiology section of Frontiers. For this, we thank all the contributors, with whom we share the same curiosity and enthusiasm in investigating this fascinating cell type.

## Author Contributions

GM wrote the first draft and edited the revisions. AR revised and contributed new concepts to the editorial. EF significantly edited and improved the final version.

## Conflict of Interest

The authors declare that the research was conducted in the absence of any commercial or financial relationships that could be construed as a potential conflict of interest.
